# Association of Adductor Pollicis Muscle Thickness and Handgrip Strength with nutritional status in cancer patients

**DOI:** 10.1371/journal.pone.0220334

**Published:** 2019-08-02

**Authors:** Katarina Papera Valente, Betullya Lucas Almeida, Thailiny Ricati Lazzarini, Vanusa Felício de Souza, Thamirys de Souza Chaves Ribeiro, Rafael Araújo Guedes de Moraes, Taísa Sabrina Silva Pereira, Valdete Regina Guandalini

**Affiliations:** 1 Department of Integrated Education, Federal University of Espirito Santo, Vitoria, Espírito Santo, Brazil; 2 University Cassiano Antônio Moraes Hospital, Vitória, Espírito Santo, Brazil; 3 Universidad de las Américas Puebla, Cholula, Puebla, México, Ex Hacienda Sta. Catarina Mártir S/N, San Andrés Cholula, Puebla, México; University of Houston, UNITED STATES

## Abstract

**Background and aim:**

Malnutrition is common in patients with cancer, and its early diagnosis can reduce or prevent further complications and improve the clinical and nutritional prognosis. Adductor Pollicis Muscle Thickness (APMT) and Handgrip Strength have been explored in this population to identify a reduction in strength and muscle mass prior to the use of conventional methods. We aimed to correlate APMT and Handgrip Strength with conventional anthropometric variables in cancer patients and verify their association with nutritional status as determined by the Patient-Generated Subjective Global Assessment (PG-SGA).

**Methods:**

A cross-sectional study was conducted with 80 patients diagnosed with cancer who were candidates for surgery. Nutritional status was obtained from the PG-SGA. Conventional anthropometric measurements were taken, as well as APMT and Handgrip Strength. Pearson’s correlation analysis and multivariate linear regression were applied to detect the influence of variables on APMT and HGS. A significance level of 5.0% was considered.

**Results:**

A high prevalence of malnutrition and the need for dietotherapic intervention was found, identified by the PG-SGA. Correlations between APMT and Handgrip Strength with anthropometric variables and with the PG-SGA score were observed. After regression adjustments, the variables that interacted with APMT were TSF and AC, and the PG-SGA score, corrected Muscle Arm Area (CAMA), and age interacted with Handgrip Strength.

**Conclusion:**

Correlations between anthropometric measurements and the PG-SGA score with APMT and Handgrip Strength were observed, even after adjusting for age and sex. These associations demonstrate that APMT and Handgrip Strength can be used with criterion in patients with cancer as complementary methods to evaluate nutritional risk and the need for nutritional intervention.

## Introduction

Malnutrition in patients with cancer is widely known as a negative prognosis factor with expressive consequences, such as a decreased response to treatment; an increased incidence of infections; increased morbidity, mortality, and length of hospitalization; decreased functional ability; and increased hospital costs [1–4], moreover, malnutrition affects 20% to 80% of the patients with cancer population [4–6].

Among the main characteristics of malnutrition in these patients, the loss of strength and muscle activity and reserve are frequent and have a negative effect on treatment and clinical results [7]. To this end, a combination of objective and subjective methods enables greater sensitivity and specificity in diagnosis, which allows the evaluation and planning of more appropriate and individualized nutritional strategies [8,9].

The Patient-Generated Subjective Global Assessment (PG-SGA) is the subjective instrument considered the most appropriate for screening and nutritional evaluation of patients with cancer [10], as it assesses different aspects, such as weight loss, dietary intake, and nutritional impact symptoms, besides enabling patient participation [8,9,11]; however, due to its subjectivity, the instrument depends on the skill of the observer [12].

As for objective methods, Adductor Pollicis Muscle Thickness (APMT) has aroused interest for its ability to assess the skeletal muscle compartment in a practical, rapid, low-cost, and non-invasive way and for its association with reduction of muscle mass and malnutrition, length of hospitalization, clinical outcomes, and mortality, as well as other methods widely used in hospital practice [13,14,15,16,17]; moreover, it enables monitoring of the muscle compartment and nutritional recovery [18].

Handgrip Strength, another objective measure, assesses strength and function and has been associated with nutritional status, since it is more influenced by changes in nutritional status than by body composition [19]. Handgrip strength has been used in several clinical conditions, including cancer, since the loss of strength, muscle mass, and functional ability follow disease progression and declining nutritional status [20,21,22,23,24].

Although some studies have proven the utility of APMT and handgrip strength in assessing malnutrition and functional ability in hospitalized patients, few studies have been conducted with cancer patients. Therefore, this study aimed to correlate APMT and handgrip strength with conventional anthropometric variables in patients with cancer and verify their association with nutritional status as determined by the PG-SGA.

## Methods

### Subjects and study period

A descriptive, cross-sectional study was conducted using convenience sampling in a tertiary referral hospital located in Vitória, Espirito Santo/ES, Brazil. Our study included patients of both sexes, aged ≥20 years, with a confirmed clinical diagnosis of cancer (ICD: C00 A C97), regardless of the type and location of the tumor, who were candidates for surgery, could be evaluated within 48 hours of hospital admission, and were able to answer the PG-SGA and perform the handgrip strength test. We evaluated those patients admitted to the General Surgery Unit from March 2017 to April 2018.

Patients were excluded if they had edema in the hands, were isolation by aerosols, could not walk independently, or did not submit complete and/or reliable information. A total of nine patients were excluded.

### Assessment of nutritional status

#### Patient-Generated Subjective Global Assessment (PG-SGA)

The PG-SGA is an instrument that assesses nutritional status and the risk of malnutrition based on weight loss, food intake, nutritional impact symptoms, functional ability, physical examination, and metabolic stress.

The PG-SGA classifies nutritional status into three categories: A = well nourished; B = suspicion of or moderate malnutrition, and C = severe malnutrition. This version also assesses, through a numerical score, the need for nutritional intervention, based on the scores obtained: 0–1 = no need for intervention, 2–3 = requires nutrition education with the patient and family, 4–8 = requires nutrition intervention, and ≥9 = requires critical nutrition intervention and control of symptoms [25].

Due to the characteristics of the study sample, the questionnaires were filled out by the subjects according to the answers given by the patients. Ratings and scores were generated by a single assessor. The version translated and validated into Brazilian Portuguese by Gonzalez et al. [25] was used, with permission to use PG-SGA/Pt-Global Platform (global.org www.pt).

#### Anthropometry and body composition

Anthropometric measurements were taken at the bedside by trained assessors. All patients were assessed in the first 48 hours of hospitalization, and the data collected were noted in an individual file. We considered current and usual weight, height, tricipital skinfold (TSF), arm circumference (AC), calf circumference (CC), and APMT. Body mass index (BMI) and corrected mid-upper arm muscle area (CAMA) were calculated. All measures were assessed three times, and the mean was used.

AC and CC measures were taken with an inelastic tape, and the individual stood in anatomical position. For TSF, we used Lange skinfold brand at 1 cm above the mid-arm. From the AC and TSF measures, MAC and CAMA were calculated. All these variables were measured according to Lohman et al. [26].

BMI was calculated from weight and height data, and the values proposed by the WHO [27] and by Lipschitz et al. [28] were used as references for adults and older adults, respectively. Individuals aged 60 years or older were considered older adults, according to the classification used in Brazil [29].

#### Adductor Pollicis Muscle Thickness (APMT)

APMT was measured by using a Lange skinfold caliper. Patients sat in a chair with both arms relaxed and the elbows at a 90-degree angle with the hands over the legs. APMT was measured by skinfold caliper with a continuous pressure of 10 g/mm^2^ in the vertex of an imaginary triangle formed by extension of the thumb and index finger [30]. The procedure was carried out on both hands three times, and the mean was used as the final value. Since there is no cutoff point defined for patients with cancer, and our sample consisted of patients who were candidates for surgery, the cutoff point of Bragagnolo et al. [13] was used for surgical patients, and thus, measures lower than 13.4 mm for the dominant APMT (DAPMT) and 13.1 mm for the non-dominant APMT (NDAPMT) indicate malnutrition.

#### Handgrip Strength

For the evaluation of handgrip strength, we used the Jamar Hydraulic Hand Dynamometer with a scale from 0 to 90 kg/f and a resolution of 2 kg/f, and both handles were adjusted in the second position. The patient was instructed to sit in a chair with arms, place the assessed arm beside the body with the elbow at a 90° angle, and lean the trunk in the chair without resting the body or receiving help from the assessor. Three measurements were performed on the dominant handgrip strength and non-dominant handgrip strength for approximately 5 seconds with a 1-minute interval between them [31]. The assessors provided motivational stimulus throughout the test. The maximum measure of both hands was considered, and the cutoff point was proposed by the European Working Group on Sarcopenia in Older People (EWGSO), according to sex (men: <27 kg/f; women: <16 kg/f) [32].

### Statistical analysis

The normality of the distribution of quantitative variables was tested using the Kolmogorov-Smirnov test. Correlations among variables were analyzed by the Pearson correlation coefficient. Multivariate linear regression was used to detect the influence of selected independent variables on APMT and handgrip strength (dependent variables) in both hands. The variables sex, AC, TSF, dominant handgrip strength, non-dominant handgrip strength, and the PG-SGA score were included in the APMT model. In the HGS model, the variables sex, age, DAPMT, NDAPMT, CAMA, AC, CC, and the PG-SGA score were included. Sex was included for both APMT and handgrip strength, due to differences in strength and muscle mass. Data were analyzed with the software SPSS 21.0. A 5.0% significance level was used for all tests.

### Ethical aspects

This study was approved by the Research Ethics Committee of the Federal University of Espirito Santo, under CAAE no. 27954014.0.0000.5060. Patients participated voluntarily and provided written informed consent.

## Results

Eighty patients were assessed, with an average age of 60.8 ± 13.5 years. Of these, 56.3% (n = 45) were men, 60.0% (n = 48) were older adults, 51.2% (n = 41) were non-white, and 76.2% (n = 61) had tumors in the gastrointestinal tract (GIT). According to the BMI, 36.3% (n = 29) of patients had eutrophy, while PG-SGA identified 60.0% (n = 48) with some degree of malnutrition (B+C). The PG-SGA score showed that 70.0% (n = 56) of patients had a score equal to or above 9 points (Table 1).

**Table 1:** Characterization of the sample studied.

Variables	Total
	n (%)
	80 (100.0)
**Sex**	
Male	45 (56.3)
Female	35 (43.7)
**Stage of life**	
Adult (< 60 years)	32 (40.0)
Older adults (≥ 60 years)	48 (60.0)
**Ethnicity**	
White	39 (48.8)
Non-White	41 (51.2)
**Tumor location**	
Gastrointestinal	61 (76.2)
Other	19 (23.8)
**Body mass index**	
Low weight	23 (28.7)
Eutrophic	29 (36.3)
Excess weight	28 (35.0)
**PG-SGA score**	
0–1 point	1 (1.2)
2–3 points	5 (6.3)
4–8 points	18 (22.5)
≥ 9 points	56 (70.0)
**PG-SGA**	
Well nourished (A)	32 (40.0)
Moderately malnourished (B)	24 (30.0)
Severely malnourished (C)	24 (30.0)

PG-SGA score: Patient-Generated Subjective Global Assessment score. PG-SGA: Patient-Generated Subjective Global Assessment.

Table 2 shows the frequency of adequacy of APMT and handgrip strength. As for the APMT of both hands, most patients were classified with malnutrition (>40.0%). Dominant handgrip strength proved suitable for most patients (60.0%). The non-dominant handgrip strength was adequate for 50.0% of the patients and inadequate for the other 50.0%.

**Table 2:** Frequency of adjustments for Adductor Pollicis Muscle Thickness and grip strength.

Variables	Dominant hand	Non-dominant hand
**APMT**	n (%)	n (%)
Normal	37 (46.2)	34 (42.5)
Reduced	43 (53.8)	46 (57.5)
**Handgrip Strength**		
Normal	58 (72.5)	49 (61.3)
Reduced	22 (27.5)	31 (38.8)

APMT: Adductor Pollicis Muscle Thickness;

Significant correlations of the DAPMT with AC, TSF, PG-SGA score, dominant handgrip strength, and non-dominant handgrip strength were found. NDAPMT was correlated significantly with the PG-SGA score, dominant handgrip strength, and non-dominant handgrip strength. As for handgrip strength, we observed significant correlations with DAPMT, NDAPMT, age, CAMA, and the PG-SGA score. Non-dominant handgrip strength was correlated significantly with DAPMT, NDAPMT, age, CAMA, AC, CC, and the PG-SGA score (Table 3).

**Table 3:** Correlations between Adductor Pollicis Muscle Thickness and Handgrip Strength with anthropometric variables, dynamometry and score of the Patient-Generated Subjective Global Assessment.

Variables	DAPMT (mm)	NDAPMT (mm)	DHGS (kg)	NDHGS (kg)
	r	r	r	r
Age (years)	−0.11	−0.11	-0.46*	-0.38*
BMI (kg/m^2^)	0.12	0.09	0.07	0.11
CAMA (cm^2^)	0.01	0.02	0.27*	0.34*
AC (cm)	0.22*	0.17	0.19	0.25*
TSF (mm)	0.28*	0.20	−0.11	-0.67
CC (cm)	0.08	0.06	0.21	0.24*
PG-SGA Score	-0.26*	-0.25*	-0.33*	-0.31*
DAPMT (mm)				
NDAPMT (mm)	0.94*			
Dominant handgrip strength (kg)	0.33*	0.33*		
Non-dominant handgrip strength (kg)	0.35*	0.32*	0.89*	

Pearson correlation;

*p < 0.05.

DAPMT; adductor pollicis muscle thickness in dominant hand; NDAPMT; adductor pollicis muscle thickness in non-dominant hand; DHGS: dominant handgrip strength; NDHGS: non-dominant handgrip strength; BMI: body mass index; CAMA: corrected arm muscle area; AC: arm circumference; TSF: tricipital skinfold; CC: calf circumference; PG-SGA score: Patient-Generated Subjective Global Assessment score.

Table 4 shows the results of linear regression for APMT of both hands. The choice to keep TSF in the NDAPMT model was made because this measure is more preserved on the non-dominant side. It is a measure used to assess energy reserve; therefore, its reduction indicates depletion of muscle reserves. For the DAPMT, after adjustment for age and sex, AC remained in the final model (β 0.61, _95%_CI 0.15–0.49, p < 0.001), explaining 54% of the measure For the NDAPMT, TSF remained in the final model (β 0.33, _95%_CI 0.02–0.34, p = 0.023), explaining 44% of value.

**Table 4:** Linear regression for the dependent variable Adductor Pollicis Muscle Thickness.

Variables	Crude		Adjusted*	
	β (95%CI)	p-value	β (95%CI)	p value
**DAPMT (mm)**			R^2^ = 0.539	
TSF (mm)	0.15 (0.35–0.26)	0.011	-0.24 (-0.57–0.79)	0.136
AC (cm)	0.22 (-0.004–0.451) 0.451)	0.054	**0.61 (0.15–0.49)**	**< 0.001**
DHGS(kg)	0.33 (0.06–0.28)	0.003	-0.001 (-0.26–0.26)	0.997
NDHGS(kg)	0.35 (0.07–0.31)	0.002	-0.24 (-0.11–0.38)	0.274
PG-SGA score	-0.26 (-0.32–0.31)	0.018	-0.14 (-0.25–0.59)	0.221
**NDAPMT (mm)**			R^2^ = 0.440	
DHGS (kg)	0.33 (0.06–0.29)	0.003	-0.16 (-0.20–0.38)	0.552
NDHGS(kg)	0.32 (0.06–-0.31)	0.004	0.05 (-0.24–0.29)	0.824
TSF (mm)	-0.20 (-0.01–0.02)	0.07	**0.33 (0.02–0.34)**	**0.023**
PG-SGA score	0.25 (0.33–0.23)	0.025	-0.20 (-0.01–0.23)	0.383

Linear regression; p < 0.05; DAPMT: adductor pollicis muscle thickness in dominant hand; NDAPMT: adductor pollicis muscle thickness in non-dominant hand; TSF: Triceps skinfold; AC: arm circumference; DHGS: dominant handgrip strength; NDHGS: non-dominant handgrip strength; PG-SGA score: Patient-Generated Subjective Global Assessment score.

*Adjusted between them for sex and age

The results of linear regression for APMT of both hands indicated that after adjustment for age and sex, the variables CAMA, PG-SGA score, and age remained in the final model, explaining 81% of result. As to the non-dominant handgrip strength, age remained in the model, explaining 77% of the measure (Table 5).

**Table 5:** Linear regression for the dependent variable gandgrip strength.

Variables	Crude		Adjusted*	
	β (95%CI)	p-value	β (95%CI)	p-value
**DHGS (kg)**			R^2^ = 0.806	
DAPMT (mm)	0.33 (0.23–1.05)	0.003	0.09 (-0.60–0.94)	0.660
DAPMT (mm)	0.32 (0.21–0.99)	0.003	0.07 (-0.60–0.85)	0.731
CAMA (cm^2^)	0.27 (0.03–0.36)	0.017	**0.16 (0.009–0.23)**	**0.034**
PG-SGA score	-0.33 (-0.70–-0.15)	0.003	**-0.19 (-0.45–-0.04)**	**0.017**
Age	-0.46 (-0.47–-0.18)	< 0.001	**-0.40 (-0.39–-0.19)**	**< 0.001**
**NDHGS (kg)**			R^2^ = 0.767	
DAPMT (mm)	0.35 (0.25–1.01)	0.002	0.28 (-0.35–1.38)	0.237
NDAPMT (mm)	0.32 (0.17–0.90)	0.004	-0.11 (-0.96–0.57)	0.617
CAMA (cm^2^)	0.34 (0.08–0.38)	0.002	0.14 (-0.12–0.32)	0.376
AC (cm)	0.25 (0.06–0.87)	0.026	0.08 (-0.57–0.88)	0.670
CC (cm)	0.24 (0.03–0.01)	0.035	0.15 (-0.17–0.76)	0.215
PG-SGA score	-0.30 (-0.63–-0.11)	0.006	-0.08 (-0.31–0.12)	0.369
Age	-0.38 (-0.39–-0.12)	0.000	**-0.29 (-0.30–-0.088)**	**0.001**

Linear regression; p < 0.05. DHGSD: dominant handgrip strength; NDHGS: non-dominant handgrip strength DAPMT: adductor pollicis muscle thickness in dominant hand; NDAPMT: adductor pollicis muscle thickness in non-dominant hand; CAMA: corrected arm muscle area; AC: arm circumference; CC: calf circumference; PG-SGA score: Patient-Generated Subjective Global Assessment score.

*Adjusted between them for sex and age.

## Discussion

The main findings of this study were a high prevalence of malnutrition, indicated by PG-SGA and APMT, and the need for dietotherapic intervention according to the PG-SGA score. Correlations of APMT and handgrip strength with classic anthropometric variables and with the PG-SGA score were observed. In the regression model, AC was associated with DAPMT and TSF with non-dominant handgrip strength when adjusted for age and gender. The dominant handgrip strength was associated with CAMA, PG-SGA score, and age; however, non-dominant handgrip strength was only associated with age.

High rates of malnutrition are found in cancer patients, mainly located in the GIT [4,10,22,33], the region most affected in this study. Our results showed 60.0% of patients with some degree of malnutrition, (B+C) by PG-SGA, which corroborates previous studies [4,5,10,22,33].

In addition to metabolic changes generated by the tumor, patients with tumors in the GIT often show increased symptoms with a nutritional impact, significant weight loss, reduction in food consumption, and reduced functional capacity, conditions that raise the PG-SGA score, which indicates the need for nutrition intervention [10,34] observed in this study.

The results were already expected due to the severity of the disease and because the patients were evaluated in a tertiary referral hospital with late diagnosis and treatment. Other factors related to the high prevalence of malnutrition and the need for nutrition intervention (score ≥9) were the advanced age of most of the group, location of cancer in the GIT, presence of inflammation, and cancer staging (the latter factors were not assessed in this study).

Malnutrition measured by objective methods was also confirmed. Significant correlations were observed between the DAPMT, AC, TSF, PG-SGA score, and handgrip strength of both hands, while the NDAPMT was correlated significantly with the PG-SGA score and handgrip strength of both hands. After the regression adjustments, the results indicated that the variables that most interacted with DAPMT and NDAPMT were AC and TSF, respectively.

These findings agree with other studies that assessed candidates for surgery [13,14,35]. APMT has been indicated as a promising measure in the diagnosis of malnutrition for being able to reveal changes in the muscle composition of the whole body, indicating early changes related to malnutrition and the recovery of nutritional status [16,26].

As to the results found after regression analysis, it is possible that they were achieved because TSF and AC are indicative of peripheral fat mass and total-body skeletal muscle mass [35,36,37], besides being measures of the same nature [13]. Patients with cancer tend to have a highly catabolic metabolism, which would result in decreased AC and TSF due to the increase in proteolysis and lipolysis, rapid weight loss, and severity of the disease.

However, APMT should not be used in isolation due to the absence of a cutoff point for this population, but as a direct measure, it has the advantages of not requiring adjustment formulas, being the only muscle that allows proper thickness evaluation for its anatomical definition, and being flat, which may facilitate nutritional evaluation by a trained assessor and its inclusion in clinical practice [15,16,30,38].

Dominant handgrip strength was associated with age, CAMA, PG-SGA score, and APMT of both hands, while non-dominant handgrip strength was correlated with age, CAMA, AC, CC score, and APMT of both hands, with age, PG-SGA score, and CAMA for dominant handgrip strength and age for non-dominant handgrip strength remaining in the model after adjustments for regression. Differences between dominant handgrip strength and non-dominant handgrip strength are expected and have already been described. In general, the dominant hand performs, on average, 10.0% better then the non-dominant hand in both sexes [39].

Handgrip strength is a frequently used, validated, non-invasive, rapid, simple, and clinical method for the measurement of muscle activity [19,20]; however, there is also a cutoff point defined for this population and standardization of the measurement technique, which can affect the comparison of results. The relationship between age and handgrip strength is already known and appears with the loss of strength and lean body mass as age progresses, causing older adults to present typically lower handgrip strength than young and middle-aged adults [40,41], which is justified in this study, since it has a larger number of older adults. Zhang et al. [21] found that the handgrip strength decreased as age increased and that the decrease in handgrip strength was twice as fast in older adults.

Although handgrip strength, PG-SGA score, and CAMA assess different parameters, they are related to strength, lean body mass, and nutritional status, since malnutrition generates changes in the muscle compartment, measured here by CAMA, and these changes can bias the estimation of functional capacity by PG-SGA, which may explain the results found. Thus, other studies have shown the association of low functionality, evaluated by handgrip strength, with nutritional status [29–31].

These findings may be influenced by reduced muscle mass and increased body fat, which occur throughout the aging process and with excess weight gain, in addition to changes in body composition in patients with cancer [42,43,44]. Reductions in muscle strength, mass, and function are usually attributable to a decrease in muscle size; however, evidence has shown a new scenario, known as myosteatosis, characterized by fat infiltration into the muscle [43,45,46].

It is possible that the increase in corporal fat reduces the capacity for muscular power generation, which is more closely related to functional capacity than muscular force [45].

This hypothesis should be considered since there was a predominance of older adults, and a significant proportion of the patients were classified as well-nourished and/or overweight by PG-SGA and BMI. Thus, the absence of a cutoff point for patients with cancer limits the interpretation of the results beyond the absence of an evaluation by computed tomography or magnetic resonance imaging that could safely indicate the body composition of the patients evaluated [47].

Our study is relevant for using the PG-SGA score, as the global score can exhibit greater interobserver variability, while the PG-SGA score is an objective and continuous method comprising the sum of all the questions.

The study has some limitations because it is transverse, includes unique measures, and includes patients with several types of tumors; therefore, it is not possible to determine the causal relationship between the variables or to extrapolate the results. Another limitation is the lack of data on cancer staging, which is because the hospital is not specialized in the treatment of patients with cancer, mainly receiving patients for surgical correction.

Another possible limitation is the use of calipers to take measurements. Discrepancies can be associated with the error at the moment the correct anatomical point is pinched, or in the calibration of the apparatus, as well as in the variability between evaluators. To correct this problem, the evaluators were well trained, and the caliper was calibrated often.

However, studies that can confirm and indicate the use of APMT and handgrip strength in surgical patients with cancer are necessary. The results found here clarify associations of APMT and handgrip strength with the instruments used in hospitals, suggesting their implementation in the clinical routine.

## Conclusion

Correlations between anthropometric measurements and the PG-SGA score with APMT and handgrip strength were observed, even after adjusting for age and sex. These associations demonstrate that APMT and handgrip strength can be used with criterion in patients with cancer and can complement the evaluation of nutritional status and the need for nutritional intervention.

However, new studies must be carried out with this population to define specific cutoff points for adults and older adults, as well as longitudinal studies to indicate causal relationships and the changes in measures of APMT and handgrip strength that occur during the hospital stay.

## Supporting information

S1 Data Data. (SAV)

S1 Table Means, medians, variance measures. (DOCX)
