# Supplementary material for: Association of Adductor Pollicis Muscle Thickness and Handgrip Strength with nutritional status in cancer patients
Source: PLoS One. 2019 Aug 2;14(8):e0220334. doi: 10.1371/journal.pone.0220334 (PMC6677294; doi:10.1371/journal.pone.0220334)
Supplement: S1 Table — (DOCX) [file pone.0220334.s002.docx]

| **S1.**  Means and standard deviations of the anthropometric variables. | | |
| --- | --- | --- |
| **Variables** | **Mean (SD)** | **Min-Max** |
| Age (years) | 60.8 (13.5) | 21.0 – 87.0 |
| BMI (kg/m^2^) | 23.8 (5.4) | 12.8 – 35.6 |
| CAMA (cm²) | 30.7 (13.2) | 7.4 – 90.6 |
| AC (cm) | 27.4 (4.9) | 17.0 – 39.0 |
| TSF (mm) | 17.4 (9.5) | 3.1 – 48.0 |
| CC (cm) | 33.0 (4.6) | 18.7 – 45.5 |
| PG-SGA Score | 13.1 (7.5) | 1.0 – 30.0 |
| DAPMT (mm) | 13.8 (5.0) | 4.9 – 36.6 |
| NDAPMT (mm) | 13.2 (5.3) | 3.7 – 40.6 |
| DHGS (kg) | 27.1 (9.7) | 11.0 – 60.0 |
| NDHGS (kg) | 24.5 (9.1) | 8.0 – 54.0 |
| DAPMT; adductor pollicis muscle thickness in dominant hand; NDAPMT; adductor pollicis muscle thickness in non-dominant hand; DHGS: dominant handgrip strength; NDHGS: non-dominant handgrip strength; BMI: body mass index; CAMA: corrected arm muscle area; AC: arm circumference; TSF: tricipital skinfold; CC: calf circumference; PG-SGA score: Patient-Generated Subjective Global Assessment score. | | |
